# Can steroidal ovarian suppression during the luteal phase after oocyte retrieval reduce the risk of severe OHSS?

**DOI:** 10.1186/s13048-015-0190-y

**Published:** 2015-09-23

**Authors:** Ya-Qin Wang, Jin Luo, Wang-Min Xu, Qin-Zhen Xie, Wen-Jie Yan, Geng-Xiang Wu, Jin Yang

**Affiliations:** Reproductive Medical center, Renmin Hospital of Wuhan University, Wuhan, Hubei 430060 China

**Keywords:** Ovarian hyperstimulation syndrome, In vitro fertilization, Luteal phase, Mifepristone, Aromatase inhibitors, Gonadotropin-releasing hormone antagonist, Prevention

## Abstract

**Background:**

Ovarian stimulation in IVF cycle results in luteal supraphysiological steroid concentrations especially for high response patients. The aim of this study was to evaluate the efficacy of ovarian steroid hormone suppression in luteal phase after oocyte retrieval for preventing severe ovarian hyperstimulation syndrome (OHSS) in high-risk patients with embryo cryopreservation.

**Methods:**

281 patients with high risk of OHSS were enrolled in this study among 4735 infertile women undergoing their first IVF treatment. The subjects were allocated into treatment and control group. The treatment group (*n* = 161) received letrozole (*n* = 43), mifepristone (*n* = 51), cetrotide (*n* = 39) and three-drug combinations (*n* = 28) during the luteal phase after oocyte retrieval, respectively. The control group (*n* = 120) received no medicine. Fertilization rate, good embryo rate, serum steroid concentration, clinical outcome, and incidence of severe OHSS were compared between the two groups.

**Results:**

On days 2, 5 and 8 after oocyte retrieval, serum estradiol levels in the letrozole and three-drug combination therapy group were significantly lower than in the other three groups at the same time (*P* < 0.001, respectively). There were no significantly difference of serum luteinizing hormone concentration on days 2, 5 and 8 and progesterone concentration on day 8 after oocyte retreival among the five groups (*P* > 0.05, respectively). Compared with the control group, the incidence of severe OHSS, the paracentesis rate, the duration of hospitalization and the days of luteal phase in each subgroup of treatment groups was not significantly decreased (*P* > 0.05, respectively).

**Conclusions:**

Our findings indicate that steroidal ovarian suppression in luteal phase after oocyte retrieval seems to be unable to prevent severe OHSS in high-risk patients with embryo cryopreservation.

## Background

Ovarian hyperstimulation syndrome (OHSS) is a serious iatrogenic and potentially life-threatening complication of induced ovulation. It is caused by an exaggerated response to gonadotropin-induced ovulation, such as the kind used during assisted reproductive technologies. The syndrome is characterized by cystic enlargement of the ovaries and an increase in capillary permeability, with the consequent acute third-space fluid sequestration and its related morbidity [1, 2]. The prevention and mitigation of the incidence and severity of severe OHSS by whatever means would be a great boon to in vitro fertilization (IVF). Despite many years of clinical experience, the pathophysiology of OHSS is still obscure. Delaying embryo transfer with embryo cryopreservation can definitely avoid pregnancy-associated late OHSS. However, there are still no precise methods of completely eliminating the incidence of human chorionic gonadotrophin (hCG)-induced early severe OHSS [3].

High estradiol (E_2_) concentrations have been associated with increasing likelihood of developing OHSS and there is some evidence to suggest that coasting can significantly reduce the incidence of severe OHSS by withdrawing exogenous gonadotropins until the patient’s serum estradiol concentration falls to a safer level before hCG trigger [4–7]. Ovarian stimulation in IVF cycle also results in supraphysiological concentrations of progesterone and estrogen during the luteal phase. In recent years, two studies reported that the addition of aromatase inhibitor for high-risk OHSS patients during the luteal phase of stimulated donor IVF cycles significantly reduces serum estradiol levels and impacts corpus luteum function [8, 9]. In animal studies, low doses of RU-486, an anti-progestin, caused alterations in ovarian weight, peritoneal capillary permeability, and the volume of ascites. These were associated with a visible decrease in OHSS in model rats [10, 11]. This raises questions about the relationship with ovarian steroid hormone levels and OHSS during the luteal phase. Whether the suppression of ovarian steroid hormone productions and promotion of regression of the corpus luteum during the luteal phase after oocyte retrieval in patients at high risk of OHSS can decrease the incidence and severity of OHSS has yet to be verified.

Gonadotropin-releasing hormone antagonist (GnRH-ant) has seen widespread use in the past two decades in in vitro fertilization-embryo transfer (IVF-ET), where it prevents the surge in luteinizing hormone (LH) surge and suppression of estradiol levels in follicular phase [12]. The use of GnRH antagonist protocol in IVF has been found to be associated with a significantly lower incidence of OHSS and E_2_ concentrations on the day of hCG administration than on the day of treatments compared with GnRH agonists [13, 14]. In recent years, luteal phase GnRH-ant administration has appeared to prevent patient hospitalization for patients with established severe early OHSS and results in quick regression of the syndrome on an outpatient basis [15–19]. However, the LH values fall rapidly after oocyte retrieval in the luteal phase of the stimulated cycles, making it unclear whether exogenous suppression of LH levels during the luteal phase is necessary? Can it really block pathogenesis of OHSS and reduce the risk of severe OHSS? For above reasons, the specific aim of this study was sought to investigate the relationship between administration of steroidal ovarian suppression in the luteal phase and OHSS.

In the present study, aromatase inhibitor, anti-progestin, and GnRH-ant were given to patients at high risk of OHSS in luteal phase after oocyte retrieval. The efficacy of intervention during the luteal phase after oocyte retrieval for the prevention and treatment of early-onset OHSS in patients undergoing embryo cryopreservation was examined.

## Methods

### Patient population

This is a prospective, observational, cohort study performed at the Reproductive Medical Center of the Renmin Hospital of Wuhan University in China from January 2010 to December 2013. A total of 281 women at high risk of OHSS among 4735 consecutive patients under 38 years old planning to undergo their first IVF treatment were included in this study. All participating patients met at least one of the following criteria: (1) Number of retrieved oocytes ≥ 20; (2) mean number of follicles with a diameter greater than 14 mm ≥ 20; (3) serum E_2_ concentrations reached ≥ 8 000 pg/ml; (4) on the day of oocyte retrieval, the ovarian diameter was > 10 cm; and\presentation of obviously symptoms of OHSS on the day of aspiration. The couples were given counseling regarding the high risks and symptoms of OHSS and all agreed to cancel fresh embryo transfer. Each patient was allowed to participate in the study only once. We strictly obeyed the Declaration of Helsinki for Medical Research involving human subjects during the project and written consent was obtained from all subjects. The protocol was approved by the Ethical Research Committee of Renmin Hospital of Wuhan University (No.WHR09125).

### Stimulation protocol and IVF procedure

In all the cases, a long mid-luteal GnRH agonist protocol was adopted for superovulation. Down-regulation was carried out using daily GnRH agonist (triptorelin, s.c.0.1 mg, Ferring, Pharmaceuticals, Kiel, Germany) beginning on day 21 of the previous cycle, as confirmed by a blood test. After 2–3 weeks of down-regulation, confirmed by a blood test and ultrasound, gonadotropin (Gonal-F, im, 75 IU or 450 IU, Merck-Serono, Aubonne, Switzerland) was administered intramuscularly at 112.5–225 IU/day starting on cycle days 5–8 of stimulation. Gn dose was adjusted according to ovarian response. All patients were monitored using transvaginal ultrasound and serum ovarian steroid hormone concentrations during superovulation. Final oocyte maturation was triggered when at least three follicles ≥ 17 mm were present on ultrasound, with administration of 6000–8000 IU hCG injection (hCG, 1000 IU, Lizhu Pharmaceuticals, Zhuhai, China). Transvaginal oocyte aspiration was performed 36 h later by ultrasound-guided follicle puncture. All embryos were cryopreserved on day 3 after IVF due to high risk of OHSS and/or severe early-developing OHSS.

### Grouping and intervention

281 consecutive high-risk patients received intra-venous fluid administration after the day of oocyte retrieval and were divided into a treatment group (*n* = 161) and control group (*n* = 120). The patients in the treatment group were informed of treatment options: letrozole group: (*n* = 43) received aromatase inhibitors letrozole tab (2.5 mg, bid, Femara; Novartis, Barcelona, Spain) per day for 5 consecutive days beginning on the day after oocyte retrieval. Mifepristone group (*n* = 51): received mifepristone tab (25 mg, bid; Zizhu Pharmaceuticals, Beijin, China) per day for 3 consecutive days beginning on the day after oocyte retrieval. GnRH-antagonist group (*n* = 39): received cetrotide (0.25 mg, qd, subcutaneous, Merck-Serono, Halle, Germany) per day for 5 consecutive days beginning on the day after oocyte retrieval. Three-drug combinations (Combinations) group (*n* = 28): took letrozole, mifepristone and cetrotide together at the same time. The control group (*n* = 120): received no special medication and was similar to the study group with regard to basal characteristics and ART stimulation parameters. In addition, 4454 patients under 38 years old planning to undergo their first IVF treatment as the non-high risk group were included and data on basal parameters were analyzed.

### Steroid hormone assay

Blood was withdrawn from patients in all groups on days 2, 5, and 8 after oocyte retrieval. Serum E_2_, LH, and progesterone (P_4_) levels were measured using an Immulite analyzer and commercially available kits (DPC, Los Angeles, CA, U.S.). Analytical sensitivity were 15 pg/ml for E_2_, 0.2 ng/ml for P_4_ and 0.1 mIU/ml for LH. Intra- and inter-assay precisions at the concentrations of most relevance to the current study (expressed as coefficients of variation) were 6.1 and 6.3 % for E_2_, 7.8 and 10.1 % for P_4_, and 5.8 and 8.2 % for LH, respectively.

### Monitoring of patients

Monitoring of patients consisted of general information, symptoms, complications during the hospitalization (ovarian torsion, thromboembolic events), embryonic condition, body mass index (BMI), abdomen circumference, ascites, and pleural effusion, urine output, days of luteal phase, whether paracentesis had taken place, and the amount of albumin (Alb) transfused were monitored and recorded. Biochemical values such as hematocrit, white blood cell count, Alb levels, blood urea nitrogen, creatinine, liver enzymes, prothrombin time, and partial thrombin time were measured when necessary. Patients were followed until menstruation.

The diagnostic criteria for OHSS were according to Golan’s classification [20]. Patients with mild OHSS presented with symptoms of mild abdominal distension and discomfort, possibly accompanied by nausea, vomiting and diarrhea, and an ovarian diameter of ≤5 cm. Moderate OHSS was defined as an aggravation of the aforementioned symptoms, associated with a weight gain of >4.5 kg, ascites identified by ultrasound examination and an ovarian diameter of 5–10 cm. Severe OHSS was defined as marked ascites and/or hydrothorax, hematocrit > 45 %, white blood cell count (WBC) >15,000/mm^3^, dyspnea, oliguria or abnormal liver function tests, and large ovaries (>10 cm maximum diameter).

### Statistical analysis

Statistical analysis was performed using SPSS 12.0 statistical software (Chicago, IL, U.S.) according to the intention to treat principle. All analyses of significance were two-sided and tested at the 5 % level. *P* < 0.05 was considered statistically significant. Continuous variables were tested if they presented normal distribution using the F-test. The results of the multiple groups were compared using the ANOVA and the comparison among groups was performed with an LSD test. Qualitative variables were assessed with the chi-squared method and Yate’s correction. In the present study, a serum P_4_ level over 60 ng/ml was taken to be 60 ng/ml exactly, because the samples were not diluted any further.

## Results

### General information

In this patient cohort, the five high-risk groups were compared for age, BMI, number of cases of polycystic ovary syndrome (PCOS), duration of infertility, baseline follicle-stimulating hormone (FSH), E_2_, duration of Gn stimulation, and Gn dose received by the patients. No significant differences were observed for any of the parameters (*P* > 0.05) (Table 1). The mean E_2_ concentration on the day of HCG administration, number of follicles with a diameter ≥14 mm, number of oocytes retrieved, fertilization rate, cleavage rate, and quality embryo rate were also comparable among the five high-risk groups. But the mean E_2_ concentration on the day of HCG administration, number of follicles with a diameter ≥14 mm, number of oocytes retrieved, the number of usable embryos was significant decreased in non high-risk group compare with each high-risk subgroup (*P* < 0.05) (Table 2).

**Table 1:** General information of OHSS high-risk and non high-risk groups

	OHSS high-risk groups (*n* = 281)					OHSS non high-risk group
	Letrozole group	Mifepristone group	GnRH-ant group	Three-drug group	Control group	
	(*n* = 43)	(*n* = 51)	(*n* = 39)	(*n* = 28)	(*n* = 120)	(*n* = 4454)
Age (year)	30.6 ± 3.5	29.7 ± 4.1	29.9 ± 4.2	30.2 ± 3.8	30.1 ± 4.0	30.9 ± 3.9
BMI (kg/m^2^)	21.8 ± 3.3	21.5 ± 4.3	21.7 ± 3.0	21.2 ± 3.5	21.4 ± 2.9	21.3 ± 3.6
PCOS (n)	7 (16.3 %)	8 (15.7 %)	5 (12.8 %)	4 (14.3 %)	18 (15.0 %)	142 (3.2 %)
Duration of infertility (year)	4.9 ± 2.5	4.7 ± 2.8	4.6 ± 3.2	4.4 ± 3.1	4.3 ± 3.5	4.8 ± 3.6
Baseline FSH (IU/L)	5.6 ± 1.8	5.3 ± 1.7	5.9 ± 1.5	5.8 ± 1.7	6.1 ± 1.6	6.3 ± 1.5
Baseline E_2_ (pg/ml)	51.2 ± 19.4	49.3 ± 17.6	50.9 ± 17.8	47.4 ± 16.5	48.2 ± 18.2	46.6 ± 18.3
Duration of Gn (days)	11.1 ± 2.0	11.2 ± 1.9	10.9 ± 1.6	11.5 ± 1.8	11.3 ± 2.1	11.2 ± 2.2
Total Gn ampoules (75 IU)	24.7 ± 6.3	24.9 ± 6.8	25.3 ± 6.5	25.7 ± 6.6	25.2 ± 6.4	28.7 ± 9.8

Values are means ± SD. Values in parentheses are percentages

*BMI* body mass index, *PCOS* polycystic ovary syndrome *FSH* follical stimulation hormone, *E*_*2*_ estradiol, *Gn* gonadotropin

**Table 2:** Treatment and in vitro fertilization parameters

	OHSS high-risk groups (*n* = 281)					OHSS non high-risk group
	Letrozole group	Mifepristone group	GnRH-ant group	Three-drug group	Control group	
	(*n* = 43)	(*n* = 51)	(*n* = 39)	(*n* = 28)	(*n* = 120)	(*n* = 4454)
Estradiol (pg/ml)^a^	7871.8 ± 2527.2	8012.4 ± 2252.6	8449.3 ± 2391.8	8225.6 ± 2734.3	8437.1 ± 2885.9	2825.6 ± 1754.2*
No. follicles with diameter >	29.2 ± 8.4	28.3 ± 7.6	30.6 ± 7.3	30.3 ± 8.1	31.1 ± 6.9	16.5 ± 6.3*
14 mm						
No. oocytes retrieved	27.4 ± 6.6	25.1 ± 5.7	27.3 ± 5.9	26.8 ± 6.2	26.4 ± 6.1	12.2 ± 6.4*
Fertilization rate (%)	82.8	80.6	81.1	79.7	83.2	79.5
Cleavage rate (%)	98.3	97.5	98.3	97.1	97.9	98.2
Quality embryo rate (%)	62.7	60.8	63.1	59.7	61.9	63.6
No. usable embryos (n)	13.2 ± 5.8	12.4 ± 4.9	13.6 ± 5.1	12.5 ± 5.6	13.7 ± 6.3	6.2 ± 2.7*

Values are means ± SD. Values in parentheses are percentages

*P < 0.01 compare with each high-risk subgroup

^a^Serum oestradiol concentration on the day of human chorionic gonadotrophin (HCG) administration

### Serum steroid hormone levels

**Fig. 1:**
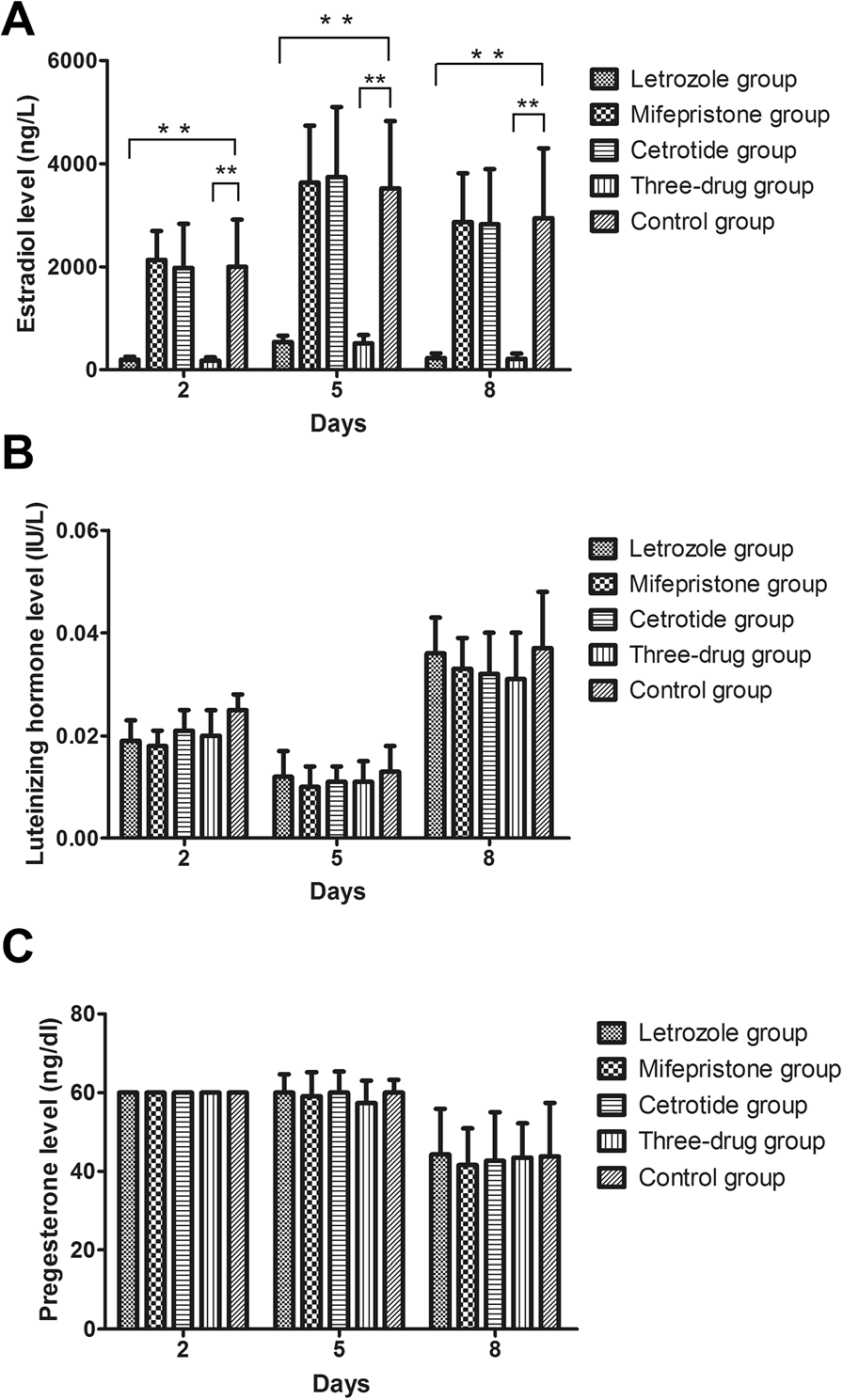
**a**, serum estradiol level; **b**, serum luteinizing hormone level; **c**, serum P_4_ level. *P < 0.05, **P < 0.001, vs. control group

**Fig. 2:**
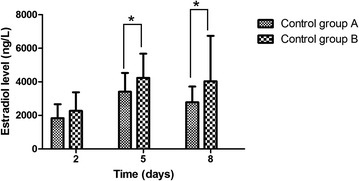
Control group **a**: mild OHSS patients; Control group **b**: moderate/ severe OHSS patients. *P < 0.05, vs. control group B

The serum E_2_, LH, and P_4_ concentrations were measured on days 2, 5, and 8 after oocyte retrieval. The results showed that, on days 2 and 5, the serum E_2_ levels tended to increase in five high-risk groups, but on day 8, there was an apparent gradual decrease (Fig. 1a). In five groups, there were no significant differences in serum E_2_ levels on days 2, 5, and 8 after oocyte retrieval within the cetrotide group, mifepristone group, and control group (*P* > 0.05), apart from a significantly lower E_2_ levels in the letrozole group and three-drug group (*P <* 0.001). The datasets of serum E_2_ levels were analyzed further on days 2, 5, and 8 in the control group. Serum E_2_ levels were significantly higher in the moderate/severe OHSS subgroup than in the mild OHSS subgroup, and the decline in E_2_ level was significantly slower (*P <* 0.05) (Fig. 2).

Serum LH levels showed that at much lower levels on days 2, 5, and 8 (LH < 0.1 IU/L) in five groups and the fluctuation was small (Fig. 1b). There were no significantly difference of serum LH concentration on days 2, 5, and 8 after oocyte retrieval in five groups (*P >* 0.05). The serum P_4_ levels were found to be >60 ng/ml on days 2 and 5, but a decrease was observed on day 8 (Fig. 1c). There were no significant differences in serum P_4_ levels on day 8 in any of the five high-risk groups (*P >* 0.05), even though the serum P_4_ values on days 2 and 5 unable to be compare accurately (because that the Clinical Laboratory of Renmin Hospital did not dilute serum samples for further testing when the serum P_4_ concentration was higher than 60 ng/ml). There were no significant differences in serum LH and P_4_ levels in any of the five groups, but serum E_2_ levels were significantly lower in the letrozole and three-drug groups than in the other groups.

### Clinical outcomes

All 281 patients were followed up until their next menses. The clinical outcomes of patients in five groups are shown in Table 3. The proportion of patients at high risk for OHSS who developed moderate early OHSS was 27.9 % (12/43), 29.4 % (15/51), 33.3 % (13/39), 25.0 % (8/28), and 27.5 % (33/120) in the letrozole group, mifepristone group, GnRH-ant group, three-drug group, and control group, respectively. The incidence of severe early OHSS in patients was 16.3 % (7/43), 19.6 % (10/51), 18.0 % (7/39), 17.9 % (5/28), and 18.3 % (22/120) in the letrozole group, mifepristone group, GnRH-ant group, three-drug group, and control group, respectively. The incidence of high risk of OHSS in this IVF population was 5.93 % (281/4735), the total incidence of severe early OHSS was 1.18 % (55/4735) while 51 out of 55 cases happened in risk patients.

**Table 3:** OHSS outcomes of high-risk treatment and control groups

	Treatment groups (*n* = 161)				Control group
	Letrozole group	Mifepristone group	GnRH-ant group	Three-drug group	
	(*n* = 43)	(*n* = 51)	(*n* = 39)	(*n* = 28)	(*n* = 120)
Paracentesis (n)	7 (16.3 %)	9 (17.7 %)	7 (17.9 %)	4 (14.3 %)	21 (19.2 %)
Length of hospital stay(days)	7.0 ± 2.9	7.4 ± 2.5	7.1 ± 2.8	6.8 ± 2.1	7.3 ± 2.7
Severity of OHSS					
Mild (n)	24 (55.8 %)	26 (51.0 %)	19 (48.7 %)	15 (53.6 %)	65 (54.2 %)
Moderate (n)	12 (27.9 %)	15 (29.4 %)	13 (33.3 %)	8 (25.0 %)	33 (27.5 %)
Severe (n)	7 (16.3 %)	10 (19.6 %)	7 (18.0 %)	5 (17.9 %)	22 (18.3 %)
Complications (n)	0	0	0	0	1^b^
Luteal phase(days)^a^	10.9 ± 2.6	10.5 ± 1.9	10.7 ± 2.4	10.4 ± 2.3	11.2 ± 3.1

Values are means ± SD unless otherwise stated. Values in parentheses are percentages. *OHSS* ovarian hyperstimulation syndrome, *NS* not statistically significant

^a^Luteal phase = interval between oocyte retrieval and next menstrual cycle

^b^One patient experienced ovarian torsion

The duration of hospitalization was 7.0 ± 2.9 days in the letrozole group, 7.4 ± 2.5 days in the mifepristone group, 7.1 ± 2.8 days in the GnRH-ant group, 6.8 ± 2.1 days in the three-drug group, and 7.3 ± 2.7 days in the control group. The incidence of moderate or severe OHSS and the duration of hospitalization was not significantly lower than in the control group in any of the treatment subgroups (*P* > 0.05). Paracentesis was performed for drainage of abdominal fluid in severe OHSS patients. In the letrozole group, 16.3 % (7/43) of the patients underwent paracentesis, 17.7 % (9/51) in the mifepristone group, 17.9 % (7/39) in the GnRH-ant group, and 14.3 % (4/28) in the three-drug group. These values are statistically similar to the 17.5 % (21/120) in the control group (*P* > 0.05).

The duration of the luteal phase (interval between oocyte retrieval and next menstrual cycle) was 10.9 ± 2.6 days in the letrozole group, 10.5 ± 1.9 days in the mifepristone group, 10.7 ± 2.4 days in the GnRH-ant group, 10.4 ± 2.3 days in the three-drug group, and 11.2 ± 3.1 days in the control group. These durations were comparable (*P* > 0.05). Notably, there were no serious adverse complications during hospitalization for OHSS observed in treatment group, but there was one case of ovarian torsion in the control group.

## Discussion

To the best of our knowledge, this is the first study to investigate the incidence of early-onset OHSS in high-risk patients using different types of steroidogenic suppression during the luteal phase. In the present study, the severe early OHSS occurred in the rate of 1.18 % for all cycles, and the incidence in high-risk patients nearly approached to 20 %, which is markedly higher than common IVF patients. This results demonstrated that the preferred management for the patients with potential OHSS included early recognition of risk factors and timely management. Ovulation induction should be highly individualized, carefully monitored, and use the minimum dose and duration of gonadotropin therapy necessary to achieve the therapeutic goal. Primary preventative measures of OHSS in follicular phase included mild ovarian stimulation, coasting, GnRH-antagonist protocol, cancel cycle, withholding HCG or GnRH agonist trigger. If the ovaries are overstimulated despite meticulous attention to the above recommendations, secondary measures should be instituted to prevent the occurrence of severe OHSS or to minimize its severity.

Ovarian stimulation in IVF cycle results in luteal supraphysiological steroid concentrations especially for OHSS high-risk patients [21–24]. The current study showed changes in serum luteal steroid concentrations on different days in patients at high risk of OHSS. Results were consistent with those of previous studies. The luteal E_2_ concentration in superovulation cycle showed the same fluctuation as in natural cycles. However, the absolute value of E_2_ was visibly higher than its natural counterparts. Serum E_2_ levels of high-risk control group with moderate/severe OHSS were significantly higher than those of patients with mild OHSS in day 2 and day 5, and the decline in E_2_ on day 8 in the former subgroup was also slower than that of the latter subgroup. This suggests to us that serum estrogen level is, to a certain degree, associated with severity of OHSS, and that caution should be exercised regarding embryo transfer in patients who show a relatively high estrogen level on day 2 or day 5 after oocyte retrieval. Moreover, P_4_ secretion was here found to very exuberant, but the peak appeared earlier, and the average duration of the luteal phase is 11.3 ± 3.0 days which is shorter than nature cycle. The results verified concept of luteolytic effect after GnRH agonist and superovulation. Furthermore, LH decreased to a very low level after aspiration as same as nature cycle. According the secretion of steroid hormone in luteal phase, to minimize the risk of severe complications, secondary preventative measures are applied by different steroidogenic suppression therapy during luteal phase for OHSS high-risk patients.

A close relationship was observed between high levels of serum estradiol and the incidence of OHSS, as reported previously by others [25–27]. After superovulation, patients at high risk of OHSS have relatively high estradiol levels not only during the follicular phase but also during the luteal phase. Letrozole is a highly specific non-steroidal, aromatase-selective inhibitor that can block the conversion of androgens to estrogen [28–30]. Letrozole at doses of 1–5 mg/day can inhibit aromatase activity by 97–99 % [31]. In the present work, results showed that E_2_ levels decreased markedly on different luteal days but there was no difference in progesterone levels after letrozole administration. This is in consistent with findings reported by Fatemi et al. and Garcia-Velasco et al. [8, 9]. Unlike the Garcia-Velasco study, we found no different in serum LH levels. Further follow-up examination did not reveal any statistically significant difference in the rates of severe OHSS, duration of the luteal phase and hospitalization period between the letrozole group and control group. The high estradiol levels observed during the luteal phase may have been caused by exuberant secretion from multiple corpus luteum after superovulation in follicular phase. Exogenous aromatase inhibitor therapy during the luteal phase cannot completely blocking OHSS in either pathogenesis or pathophysiology.

Previous observations reported that elevated plasma concentrations of progesterone, in addition to estradiol, during the clinical phase of OHSS [32]. In clinical manifestation, the peak days of early OHSS coincide with maximum secretion by the corpus luteum and instantly relieves after menstruation. Severe OHSS is frequently associated with pregnancy, which suggests that progesterone is involved in OHSS pathophysiology. Mifepristone, a progesterone antagonist, is a synthetic steroid hormone that binds to the progesterone receptor. It has been predominantly and successfully used in the termination of early pregnancies [33, 34]. Ujioka et al. reported that low doses of RU-486 (<10 mg/kg) may decrease ovarian and peritoneal capillary permeability in hyperstimulated rats, probably through the anti- glucocorticoid effect and antiprogestational activity and modulation of vascular endothelial growth factor (VEGF) and different cytokine effects [10, 11]. In a recent study, Yung et al. found out that RU-486 represses LHCGR expression and LH/hCG signaling in cultured luteinized human mural granulosa cells [35]. In the current study, mifepristone was first administered during the early luteal phase for patients at high risk of OHSS who had cryopreserved all embryos. The current observation shows that steroid levels, severe OHSS rates and paracentesis rate are not significantly different in the mifepristone and control groups.

LH plays a crucial role in the steroidogenic activity of the corpus luteum that takes place during the luteal phase [36, 37]. OHSS is usually observed several days after hCG trigger, and its severe form is frequently associated with pregnancy or hCG supperly during the luteal phase [20]. LH and hCG share the same α-subunit and 81 % of the amino-acid residues of the β-subunit. They also bind to the same receptor, the LH/hCG receptor. It has been reported that luteal GnRH antagonist administration in patients with established severe early OHSS appears to prevent the need for patient hospitalization and causes quick regression of the syndrome on an outpatient basis [15–19]. Mais et al. found that administration of GnRH antagonist during the mid-luteal phase in natural menstrual cycles could induce luteolysis by reducing pulsatile gonadotrophin stimulation. This caused a rapid decline in serum estradiol and progesterone concentrations and the onset of menstrual bleeding [38]. The present study, on the contrary, showed that the luteal-phase steroid concentration is physiologically comparable to that of the control group and the incidence of severe OHSS was roughly the same in the cetrotide group and control group. These results may be explained by several factors. First, luteal phase LH secretion is profoundly suppressed by the pituitary via negative feedback actions at the level of the hypothalamic-pituitary axis because of luteal supraphysiological concentrations of progesterone and estradiol [22–24]. Cetrotide’s anti-steroid activity is weak on deeply suppressed LH levels. However, even though it has been demonstrated that a few GnRH receptors are expressed in the ovary, low-dose GnRH-ant exerts only a slight an antagonistic effect there [39].

In our study, letrozole, mifepristone, and cetrotide were co-administered and synergistic effects were observed. We found that luteal steroidogenic suppression even by three-drug combinations can’t block the ongoing process of OHSS compared with control group. Consideration should be given to the possibility that the overstimulated ovaries, provoked by exogenous hCG injection, leads to the ovarian release of vasoactive-angiogenic substances increasing the vascular permeability and the full blown syndrome as a consequence. Anti-steroidogenic thoerpy during the luteal phase can not fundamentally block the effect of hCG and pathophysiology of OHSS.

The major limitation in this pilot cohort study was the lack of information regarding the VEGF, interleukins and rennin angiotensinogen system which have been implicated in the pathogenesis of OHSS in the serum and ascites of these patients. This study is also limited by its sample size of subgroup and non-randomized design. No experiments were performed to compare the effects of these drugs at different dosages, the timing or duration of treatment, or different types of anti-steroidogenic medicine.

## Conclusions

These data demonstrate that, at present, there is no difference in the rate of severe OHSS rate in patients at high risk of OHSS, regardless of the type of luteal steroidogenic suppression given. Currently, the etiology of OHSS is not sufficiently known and therapy remains experimental. In patients at high risk of OHSS, once triggered by hCG, OHSS induces a cascade of effects. Steroidogenic suppression during the luteal phase does not achieve a satisfactory outcome. The key to the primary prevention of OHSS during control ovarian stimulation is recognition of risk factors and individualization of the ovarian stimulation protocol. Further studies and more patients are needed to determine how feasible it is to completely eliminate OHSS.

## Abbreviations

BMI: Body mass index
E_2_: Estradiol
FSH: Follicle-stimulating hormone
GnRH-ant: Gonadotropin-releasing hormone antagonist
hCG: Human chorionic gonadotrophin
IVF-ET: In vitro fertilization-embryo transfer
LH: Luteinizing hormone
OHSS: Ovarian hyperstimulation syndrome
P_4_: Progesterone
PCOS: Polycystic ovary syndrome
VEGF: Vascular endothelial growth factor
